# Sarcopenic visceral obesity in patients with metabolic dysfunction-associated steatotic liver disease (MASLD)

**DOI:** 10.1007/s10238-025-01865-y

**Published:** 2025-10-21

**Authors:** Maha Elsabaawy, Amr Ragab, Amal Abd-Elrazek, Mohamed Atef, Madiha Naguib

**Affiliations:** 1 Department of Hepatology and Gastroenterology, National Liver Institute, Menoufia University, Shebeen Elkoom, Menoufia Egypt; 2 Department of Radiodiagnosis and Interventional Radiology, National Liver Institute, Menoufia University, Shebeen Elkoom, Menoufia Egypt

**Keywords:** Sarcopenic obesity, MASLD, MRI, Visceral fat, Liver fibrosis, Cardiovascular risk, Tier classification, Body composition

## Abstract

Sarcopenic visceral obesity (SVO) has emerged as a high-risk metabolic phenotype in metabolic dysfunction-associated steatotic liver disease (MASLD). This study aimed to define the prevalence and metabolic implications of MRI-defined SVO in MASLD, evaluate its association with liver fibrosis, cardiovascular risk, and introduce a novel tier-based classification for risk stratification. In this cross-sectional study, 334 adults with MASLD underwent comprehensive phenotyping. Sarcopenia was assessed by bioelectrical impedance analysis, while visceral obesity was quantified via MRI-based visceral fat area (VFA ≥ 100 cm^2^). Liver fibrosis was evaluated using non-invasive indices and confirmed in a subset by magnetic resonance elastography (MRE). Participants were stratified into SVO and non-SVO groups, and further categorized into Red, Yellow, or Green tiers based on fibrosis stage and cardiovascular risk. SVO was present in 42.5% of MASLD patients, with higher prevalence among women and individuals with BMI ≥ 40. SVO was associated with significantly worse metabolic profiles (HOMA-IR: 6.2 ± 2.8, *p* < 0.001), advanced fibrosis (FIB-4: 2.3 ± 1.4, *p* = 0.003), and higher cardiovascular risk (ASCVD ≥ 7.5%: 65%, *p* < 0.001). In multivariate analysis, SVO independently predicted advanced fibrosis (OR = 2.5, *p* = 0.002). Importantly, a tier-based classification model identified a high-risk “Red Tier” group (100% F3–F4 fibrosis, 100% diabetes). This is the first study in a Middle Eastern MASLD cohort to combine MRI-based adiposity assessment with validated sarcopenia criteria to define SVO and demonstrate its prognostic relevance. The introduction of a tiered risk framework integrating SVO, fibrosis, and ASCVD risk represents a novel approach to personalized MASLD care and support targeted decision-making.

## Introduction

Metabolic dysfunction-associated steatotic liver disease (MASLD), the newly designated umbrella term for nonalcoholic fatty liver disease (NAFLD), has become a leading cause of chronic liver disease globally [1]. Its pathogenesis is intricately linked to a constellation of metabolic abnormalities, including insulin resistance, visceral adiposity, and chronic low-grade inflammation [1]. Amidst these risk factors, a distinctive and increasingly recognized body composition phenotype—sarcopenic visceral obesity (SVO)—has emerged as a potent contributor to metabolic and hepatic deterioration [2].

SVO, characterized by the concurrent presence of sarcopenia (loss of skeletal muscle mass and function) and visceral obesity, reflects a state of dual anabolic resistance and ectopic fat accumulation. This combination generates a metabolically adverse milieu, amplifying insulin resistance, systemic inflammation, and lipotoxic stress—factors central to MASLD progression. Importantly, while sarcopenia and obesity have historically been investigated as independent entities, contemporary evidence suggests that their intersection exerts a compounded effect, predisposing individuals to more aggressive hepatic phenotypes and worse cardiovascular outcomes [3–5].

Despite these insights, the epidemiological footprint and pathophysiological consequences of SVO remain poorly defined in MASLD—particularly in regions such as the Middle East and North Africa, where metabolic diseases are rapidly escalating yet remain underrepresented in global datasets [6]. Existing studies have also been hampered by imprecise definitions and reliance on surrogate markers [7–9]. For instance, many assessments of visceral obesity depend on waist circumference or BMI, which fail to differentiate between subcutaneous and visceral fat. Similarly, muscle mass estimation often lacks standardization [7–9]. To overcome these limitations, advanced imaging tools such as magnetic resonance imaging (MRI) and magnetic resonance elastography (MRE) now offer precise, non-invasive quantification of body composition and liver fibrosis—ushering in a new era of phenotypic stratification in MASLD.

This study aims to comprehensively characterize SVO in patients with MASLD using state-of-the-art imaging and consensus sarcopenia definitions (ESPEN/EASO 2022), providing an integrated framework that captures both metabolic and hepatic dimensions of this high-risk phenotype. By evaluating SVO prevalence, metabolic correlates, and its predictive value for advanced fibrosis—measured via validated non-invasive indices and MRE—we offer novel insights into its pathogenic role. To our knowledge, this is the first study in a Middle Eastern MASLD population to operationalize a rigorously defined SVO phenotype using MRI-based body composition profiling and elastography-confirmed fibrosis staging. Our findings aim to bridge a critical knowledge gap and propose SVO as a distinct, clinically actionable target in MASLD risk stratification and management.

## Methods

### Study design and population

This cross-sectional, observational study was conducted between January 2023 and June 2024. The study enrolled consecutive adult patients (≥ 18 years) diagnosed with metabolic dysfunction-associated steatotic liver disease (MASLD) according to the 2023 multi-society Delphi consensus criteria [1]. Exclusion criteria included secondary causes of liver steatosis—such as viral hepatitis (HBV, HCV), significant alcohol consumption (> 20 g/day), autoimmune liver diseases (e.g., autoimmune hepatitis, primary biliary cholangitis), which were ruled out via standard serologic testing (ANA, AMA, ASMA, anti-LKM1)—pregnancy, malignancy, recent hospitalization (< 4 weeks), severe cardiopulmonary comorbidities (NYHA class III/IV), and neuromuscular disorders affecting muscle mass assessment. All participants provided written informed consent, and the study protocol was approved by the institutional review board of NLI (Approval No. 0014014FWA00034015), adhering to the ethical standards of the Declaration of Helsinki.

### Clinical and laboratory assessment

Standardized anthropometric measurements were obtained by trained clinicians, including height, weight, and waist circumference, following WHO protocols [10]. Body mass index (BMI) was calculated as weight (kg)/height^2^ (m^2^), and obesity was classified according to WHO thresholds. Blood pressure was measured in a seated position using an automated Oscillometric device (Omron HEM-7320).

Biochemical parameters were analyzed after overnight fasting, including fasting plasma glucose, insulin, liver enzymes (AST, ALT), lipid profile, and hemoglobin A1c (HbA1c). Insulin resistance was quantified using the Homeostatic Model Assessment for Insulin Resistance (HOMA-IR), calculated as:

HOMA-IR = [Fasting Insulin (μU/mL) × Fasting Glucose (mmol/L)] / 22.5 Matthews et al., 1985.

The Fibrosis-4 Index (FIB-4) was calculated using the established formula involving age, AST, ALT, and platelet count to estimate liver fibrosis non-invasively [11].

### Assessment of sarcopenia and visceral obesity

#### Sarcopenia assessment

Skeletal muscle mass was assessed using multi-frequency bioelectrical impedance analysis (BIA) via the InBody 770 system, a validated, reproducible tool suitable for clinical and research settings [12]. Sarcopenia was defined per ESPEN/EASO 2022 consensus criteria as appendicular lean mass (ALM) indexed to height^2^ with cut-offs of:

-  < 7.0 kg/m^2^ for men
-  < 5.5 kg/m.^2^ for women [3]

#### Visceral adiposity assessment

Visceral adipose tissue (VAT) was quantified using magnetic resonance imaging (MRI) at the L4–L5 intervertebral level, which provides precise segmentation and volume analysis of visceral fat compartments. Visceral obesity was defined as a visceral fat area (VFA) ≥ 100 cm^2^, in accordance with prior imaging-based literature [13].

#### SVO definition

**Fig. 1:**
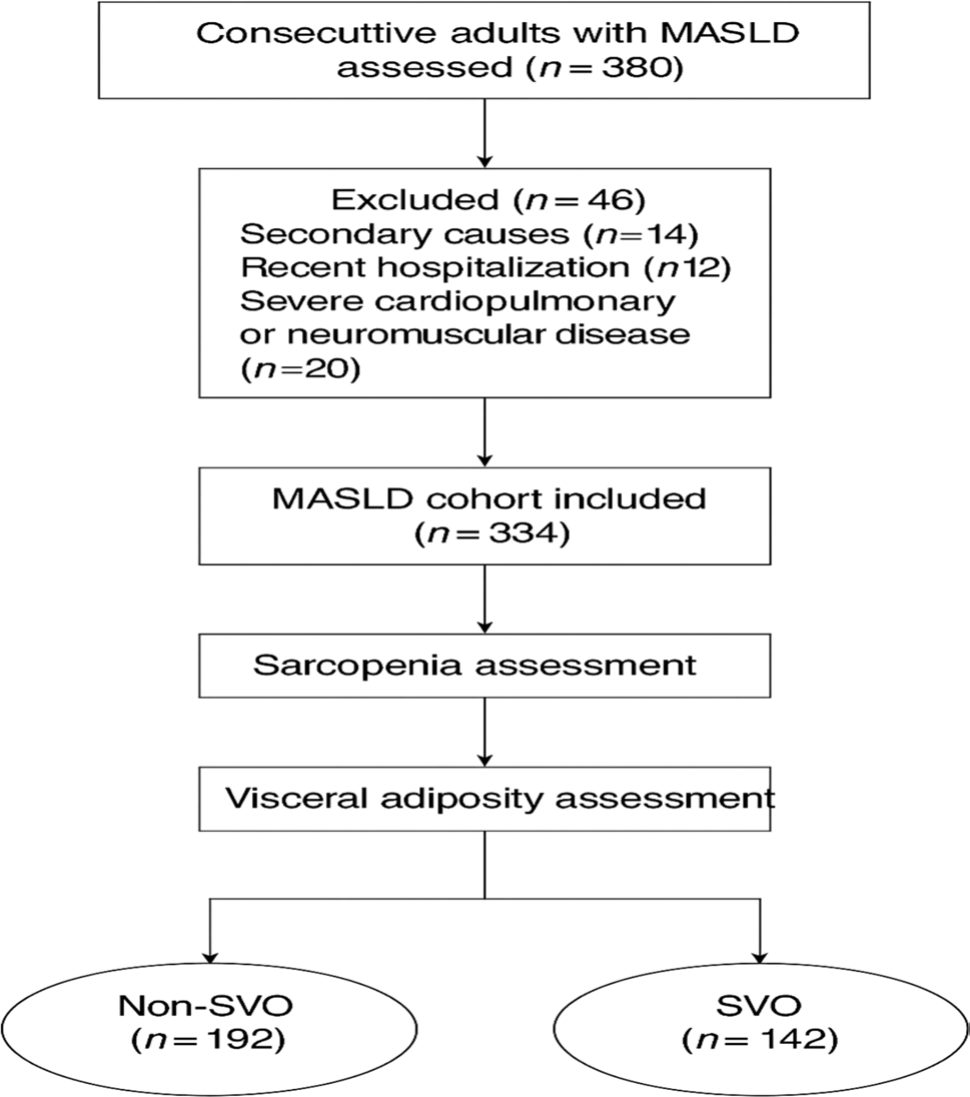
Flowchart of study cohort stratification based on metabolic burden in MAFLD. This figure illustrates the selection and classification process of the study population (N = 357) diagnosed with Metabolic Dysfunction-Associated Fatty Liver Disease (MAFLD). Patients were stratified into three distinct subtypes based on the number and type of metabolic risk factors present: Subtype 1 (n = 110; 30.8%): Presence of one metabolic criterion (either obesity, T2DM, or metabolic dysregulation). Subtype 2 (n = 161; 45.1%): Presence of two metabolic criteria. Subtype 3 (n = 86; 24.1%): Fulfillment of all three MAFLD diagnostic criteria (obesity, T2DM, and metabolic dysregulation). This classification was used for comparative analysis of hepatic fibrosis (via FIB-4 index) and steatosis (via Fatty Liver Index), with subtype 3 showing the highest burden of hepatic injury. The flowchart also reflects exclusion criteria, and the final sample used for analysis

SVO was operationally defined as the coexistence of sarcopenia and visceral obesity, satisfying both criteria described above. This dual-phenotype construct reflects a high-risk metabolic and inflammatory state, previously linked to adverse hepatic and cardiovascular outcomes (Fig. 1).

#### Liver fibrosis evaluation

Advanced fibrosis was assessed using a dual approach:

- *FIB-4 score* (≥ 2.67 considered suggestive of advanced fibrosis)
- *Magnetic Resonance Imaging (MRI) and Elastography (MRE)*

All participants underwent hepatic magnetic resonance imaging (MRI) and magnetic resonance elastography (MRE) using a standardized protocol. Liver fat quantification was assessed using proton density fat fraction (PDFF)-based MRI, while liver stiffness was measured using MRE. The MRI/MRE examinations were performed using a 1.5-Tesla or 3.0-Tesla scanner with a phased-array surface coil. Liver stiffness values were obtained from the right hepatic lobe using elastograms with confidence maps and expressed in kilopascals (kPa). Stiffness thresholds were used to stratify patients according to fibrosis stage (e.g., F0–F4), with ≥ 2.9 kPa indicating advanced fibrosis (≥ F2). All images were independently reviewed by experienced radiologists blinded to the clinical data [5].

#### Cardiovascular risk stratification

The Atherosclerotic cardiovascular disease (ASCVD) risk was calculated using the 2013 ACC/AHA pooled cohort equations, with a 10-year risk ≥ 7.5% considered high risk [14]. Multivariable models examined the independent contribution of SVO to cardiovascular risk.

#### Risk tier classification

To enable integrative risk profiling, participants were stratified into three risk tiers based on the presence of SVO, liver fibrosis stage, and ASCVD risk:

- *Red Tier (High Risk)*: Patients with SVO, advanced fibrosis (F3–F4), and a 10-year ASCVD risk ≥ 7.5%.
- *Yellow Tier (Moderate Risk)*: Patients with SVO and either advanced fibrosis or elevated ASCVD risk (but not both).
- *Green Tier (Low Risk)*: Patients with SVO alone, without evidence of significant fibrosis or elevated cardiovascular risk.

This tiered framework was developed to reflect composite hepatic–cardiometabolic vulnerability, facilitating phenotypic clustering for clinical triage. Fibrosis staging was based on FIB-4 scores and magnetic resonance elastography (MRE) data where available. ASCVD risk was calculated using the 2013 ACC/AHA pooled cohort Eqs. 14. Stratification was evaluated for associations with clinical and biochemical variables to validate internal construct integrity.

#### Sample size justification

The minimum required sample size was estimated based on the ability to detect a significant difference in fibrosis prevalence between SVO and non-SVO groups, assuming an effect size (Cohen’s *d*) of 0.4, α = 0.05, and power (1–β) = 0.90. Based on prior studies in NAFLD and sarcopenic obesity cohorts [4], a total of at least 264 participants was required. Accounting for potential dropouts and missing data, we targeted a final sample size of 330–350 patients, yielding sufficient power for multivariable modeling and subgroup analysis (e.g., by sex and obesity class). The final cohort of 334 MASLD patients met this criterion, ensuring statistical robustness.

## Statistical analysis

Data was analyzed using R version 4.3.0 and IBM SPSS Statistics version 28.0. Continuous variables were presented as mean ± standard deviation or median (IQR) and compared using Student’s *t*-test or Mann–Whitney U test, as appropriate. Categorical variables were analyzed using Chi-square or Fisher’s exact test. Logistic regression models assessed predictors of advanced fibrosis and cardiovascular risk, adjusting for potential confounders including age, sex, BMI, diabetes, lipid levels, and smoking. Multicollinearity was assessed using variance inflation factors (VIF). Spearman’s rank correlation explored associations between SVO components and metabolic/fibrotic markers. A two-sided *p*-value < 0.05 was considered statistically significant.

## Results

### Prevalence and clinical characteristics of SVO

**Fig. 2:**
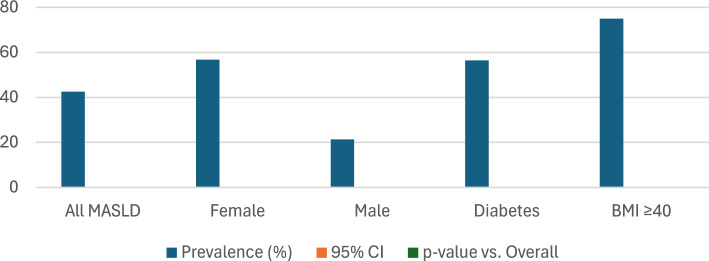
SVO Prevalence and Correlation in MASLD

Among the 334 MASLD patients enrolled, sarcopenic visceral obesity (SVO) was identified in 42.5% (*n* = 142) of the cohort. The prevalence of SVO was significantly higher among women (56.7% vs. 21.3%, *p* = 0.002) and individuals with BMI ≥ 40 (75.0%, p < 0.001), highlighting its association with both sex and obesity class (Fig. 2).

Compared to non-SVO patients, individuals with SVO were significantly older (58 ± 10 vs. 53 ± 9 years, p = 0.02), had higher BMI (38.5 ± 6.2 vs. 34.1 ± 5.9 kg/m^2^, *p* < 0.001), and demonstrated worse metabolic parameters. This included elevated insulin resistance as measured by HOMA-IR (6.2 ± 2.8 vs. 3.1 ± 1.9, *p* < 0.001), higher LDL cholesterol (128 ± 35 vs. 102 ± 29 mg/dL, *p* < 0.001), and increased triglyceride levels (198 ± 72 vs. 132 ± 58 mg/dL, *p* < 0.001). Moreover, high cardiovascular risk (ASCVD risk ≥ 7.5%) was more common in the SVO group (65% vs. 22%, *p* < 0.001), with a significantly higher mean ASCVD risk score (14.2 ± 3.8% vs. 5.1 ± 2.6%, *p* < 0.001) (Table 1).

**Table 1:** Baseline characteristics by SVO status

Variable	SVO	Non-SVO	p-value
Age (years)	58 ± 10	53 ± 9	0.02
Female sex	72%	48%	0.002
BMI (kg/m^2^)	38.5 ± 6.2	34.1 ± 5.9	< 0.001
Diabetes	85%	70%	0.06
HOMA-IR	6.2 ± 2.8	3.1 ± 1.9	< 0.001
FIB-4	2.3 ± 1.4	1.6 ± 0.9	0.003
VFatL	15.2 ± 3.1	8.7 ± 2.4	< 0.001
High ASCVD Risk (≥ 7.5%)	65%	22%	< 0.001
Mean ASCVD Risk (%)	14.2 ± 3.8	5.1 ± 2.6	< 0.001
LDL (mg/dL)	128 ± 35	102 ± 29	< 0.001
Triglycerides	198 ± 72	132 ± 58	< 0.001
Females Only			
Triglycerides	185 ± 68	121 ± 53	< 0.001
Males Only			
LDL	135 ± 38	108 ± 31	0.003
- BMI > 30 (%)	92%	45%	< 0.01
- Diabetes (%)	88%	34%	< 0.001
- Hypertension (%)	76%	38%	< 0.01
- Smoking (%)	41%	18%	0.03
Fibrosis Stage (F3-F4) by MRE	58%	12%	< 0.001

Abbreviations: **SVO**, sarcopenic visceral obesity; **BMI**, body mass index; **HOMA-IR**, homeostatic model assessment of insulin resistance; **FIB-4**, fibrosis-4 index; **VFatL**, visceral fat level; **ASCVD**, atherosclerotic cardiovascular disease; **LDL**, low-density lipoprotein cholesterol; **mg/dL**, milligrams per deciliter; MRE, magnetic resonance elastography

**Fig. 3:**
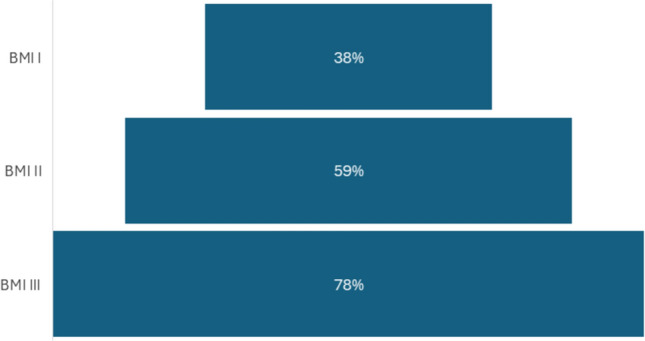
SVO across obesity classes

The prevalence of SVO increased progressively with obesity severity, peaking at 75% among patients with BMI ≥ 40, indicating a strong dose–response relationship between obesity class and SVO risk (Fig. 3).

### Fibrosis and hepatic injury

**Fig. 4:**
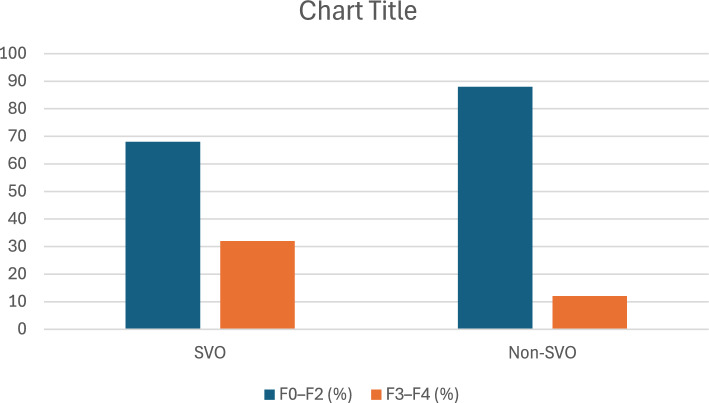
prevalence of SVO in different grades of fibrosis by MRE

SVO was strongly associated with more advanced liver fibrosis. The FIB-4 score was significantly higher among SVO patients (2.3 ± 1.4 vs. 1.6 ± 0.9, *p* = 0.003). Magnetic resonance elastography (MRE) revealed that 58% of SVO individuals had advanced fibrosis (F3–F4), in contrast to 12% in the non-SVO group (*p* < 0.001), as illustrated in Fig. 4.

**Fig. 5:**
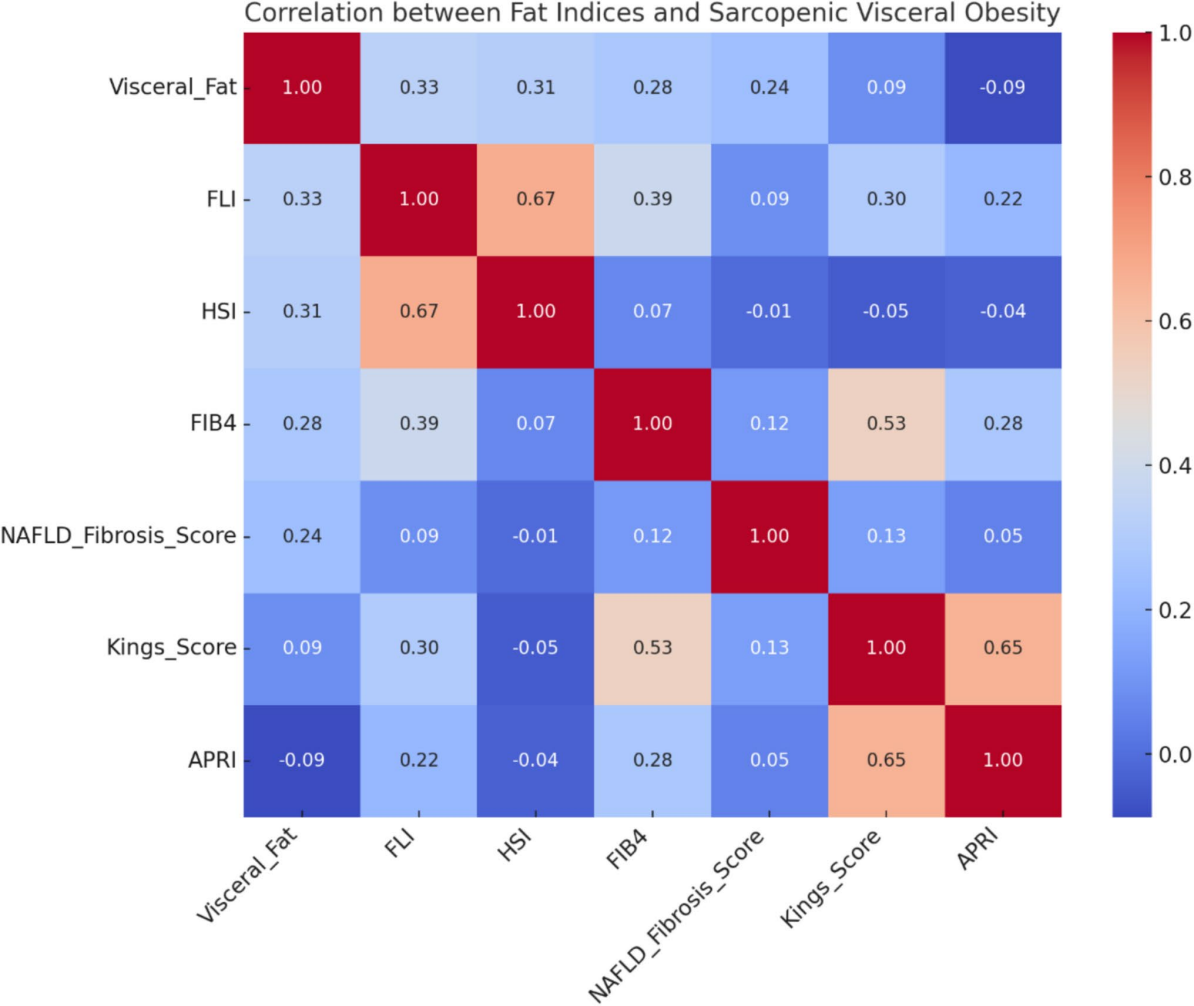
Correlation matrix between SVO and various hepatic fat and fibrosis indices

Significant positive correlations were observed between visceral fat and FLI (*r* = 0.52), HSI (*r* = 0.44), and FIB-4 (*r* = 0.28), indicating that increased visceral adiposity is strongly associated with hepatic steatosis and fibrotic burden. Conversely, the SO Index showed a negative correlation trend with these indices, reflecting the metabolic impact of muscle loss. All variables are fully labeled in the figure, and the heatmap allows for clear visual interpretation of the strength and direction of associations (Fig. 5).

Multivariate logistic regression identified SVO as an independent predictor of advanced fibrosis (adjusted OR = 2.5, 95% CI: 1.4–4.3, *p* = 0.002), along with diabetes (OR = 1.89, p = 0.006) and female sex (OR = 1.72, *p* = 0.02) (Table 2).

**Table 2:** Predictors of advanced fibrosis

Predictor	OR	95% CI	p-value
SVO	2.5	1.4–4.3	0.002
Age	1.05	0.98–1.12	0.18
Female sex	1.72	1.10–2.70	0.02
Diabetes	1.89	1.20–2.98	0.006

SVO, sarcopenic visceral obesity; OR, adjusted odds ratio; CI, confidence interval

### Cardiovascular risk and metabolic correlates

Correlation analyses revealed that the SVO phenotype significantly correlated with adverse cardiometabolic markers, including HOMA-IR, triglycerides, LDL, and FIB-4 scores (Fig. 5). In multivariate modeling, SVO emerged as the strongest independent predictor of elevated cardiovascular risk (ASCVD ≥ 7.5%), with an adjusted OR = 3.8 (95% CI 2.1–6.9, p < 0.001). This exceeded the predictive value of male sex (OR = 1.9, p = 0.02), diabetes (OR = 2.7, *p* = 0.001), and LDL > 100 mg/dL (not statistically significant) (Table 3).

**Table 3:** Predictors of high cardiovascular risk

Variable	OR (95% CI)	p-value
SVO (yes vs no)	3.8 (2.1–6.9)	< 0.001
Male gender	1.9 (1.1–3.3)	0.02
Diabetes	2.7 (1.5–4.8)	0.001
LDL > 100 mg/dL	1.6 (0.9–2.8)	0.09
Triglycerides > 150	2.1 (1.2–3.7)	0.01

Adjusted for: Age, BMI, diabetes, hypertension, smoking, fibrosis stage, LDL, triglycerides Key Finding: After full adjustment, SVO nearly quadruples ASCVD risk (aOR = 3.8), stronger than traditional risk factors like elevated LDL

### Tier-based risk stratification

Participants were further stratified into three risk categories—Red, Yellow, and Green—based on SVO status, fibrosis stage, and cardiovascular risk (Table 4). The Red Tier, representing the highest-risk group, included patients with F3–F4 fibrosis, SVO, and ASCVD risk ≥ 7.5%. All Red Tier patients also had diabetes and elevated visceral fat levels. This group had significantly higher ALT, AST, and lower ALM/weight ratios (all *p* < 0.01). Predictors of Red Tier status included advanced fibrosis (OR = 12.5, *p* < 0.001), male sex (OR = 3.2, *p* = 0.01), and elevated ALT (OR = 1.05, *p* = 0.02) (Table 5).

**Table 4:** Risk stratification by tier classification

Variable	Red (High Risk) (n = 9)	Yellow (Moderate Risk) (n = 16)	Green (Low Risk) (n = 15)	*p*-value
Age (years)	54.2 ± 9.1	53.1 ± 8.3	50.7 ± 8.9	0.45
Gender (Female)	33.3% (3/9)	75.0% (12/16)	80.0% (12/15)	0.03*
BMI	34.6 ± 6.2	39.1 ± 6.8	32.5 ± 7.1	0.02*
ALM/Weight (%)	28.9 ± 5.1 (M: < 29.6, F: < 23.4)	22.1 ± 3.2 (M: < 29.6, F: < 23.4)	23.0 ± 2.8 (M: < 29.6, F: < 23.4)	0.01*
VFatL (%)	15.6 ± 3.1 (> 12%)	14.2 ± 2.8 (> 12%)	9.1 ± 3.5 (< 12%)	< 0.001*
DM (Yes)	100% (9/9)	100% (16/16)	100% (15/15)	1.00
HTN (Yes)	55.6% (5/9)	56.3% (9/16)	13.3% (2/15)	0.01*
Smoking (Yes)	44.4% (4/9)	18.8% (3/16)	0% (0/15)	0.01*
Fibrosis Stage (F3-F4)	100% (9/9)	0% (0/16)	0% (0/15)	< 0.001*
AST (U/L)	42.1 ± 18.3	28.4 ± 10.2	24.7 ± 12.5	0.02*
ALT (U/L)	51.2 ± 25.6	30.8 ± 12.4	25.3 ± 14.1	0.01*
HbA1c (%)	7.8 ± 1.5	7.2 ± 1.8	6.5 ± 1.6	0.08
LDL (mg/dL)	148.3 ± 42.1	162.8 ± 38.5	155.2 ± 41.2	0.65

Abbreviations: BMI, body mass index; ALM, appendicular lean mass; VFatL, visceral fat level; DM, diabetes mellitus; HTN, hypertension; AST, aspartate aminotransferase; ALT, alanine aminotransferase; HbA1c, hemoglobin A1c; LDL, low-density lipoprotein cholesterol; U/L, units per liter; F, female; M, male. A risk stratification system based on specific clinical criteria. The Red tier represents the highest risk group, defined by the presence of symptomatic varicose veins (SVO), an ASCVD risk ≥ 7.5%, and fibrosis stage F3-F4. The Yellow tier includes patients with SVO and one additional comorbidity, indicating moderate risk. Finally, the Green tier consists of individuals with SVO alone, suggesting the lowest risk among the three categories. This stratification helps guide clinical decision-making by identifying patients who may require more intensive monitoring or intervention

**Table 5:** Predictors of red tier classification

Variables	Odds Ratio (OR)	95% CI	p-value
Fibrosis (F3-F4)	12.50	4.80–32.60	< 0.001
Male Gender	3.20	1.40–7.30	0.01
ALT	1.05	1.01–1.09	0.02

Abbreviations: OR odds ratio; CI, confidence interval; ALT, alanine aminotransferase

## Discussion

This study provides compelling evidence that SVO constitutes a high-risk clinical phenotype within the spectrum of MASLD. Using a rigorously defined diagnostic framework—integrating ESPEN/EASO 2022 consensus criteria for sarcopenia with MRI-quantified visceral adiposity—we identified SVO in over 42% of MASLD patients, a prevalence notably higher than reported in previous Western cohorts reliant on anthropometric proxies [3]. Our findings not only establish SVO as a prevalent entity but also as an independent predictor of advanced hepatic fibrosis and cardiovascular risk, with implications that reverberate across hepatology, endocrinology, and preventive cardiology.

SVO may potentially develop MASLD progression through interconnected metabolic pathways. Loss of skeletal muscle reduces insulin sensitivity and diminishes secretion of protective myokines such as irisin and IL-15, impairing glucose metabolism and promoting systemic inflammation [15]. Concurrently, excess visceral fat drives hepatic lipotoxicity through the release of pro-inflammatory adipokines (e.g., leptin, resistin) and cytokines like TNF-α and IL-6, which contribute to hepatocellular injury and fibrogenesis [16, 17]. This dual burden creates a metabolic milieu that accelerates liver disease progression and worsens outcomes in MASLD.

Hepatic fibrosis is a well-established prognostic marker across chronic liver diseases—not only in MAFLD, but also in autoimmune hepatitis, viral hepatitis, and alcoholic liver disease—where fibrosis stage consistently predicts treatment response, clinical decompensation, and mortality [18–20]. These cross-etiological patterns underscore the central role of fibrosis and support its routine non-invasive assessment in all liver disease frameworks.

The nearly twofold elevation in FIB-4 scores and fivefold increase in advanced fibrosis (F3–F4) among SVO patients in our cohort reinforce the synergistic role of sarcopenia and visceral obesity in liver fibrogenesis, consistent with machine learning-based survival models from cirrhosis cohorts [5].

Our data also highlight sex-based differences in SVO expression, with a significantly higher prevalence in women—an observation that may reflect hormonal modulation of fat deposition and muscle metabolism, particularly post-menopause. Furthermore, the dose–response relationship between obesity class and SVO prevalence, peaking at 75% in class III obesity, underscores the importance of evaluating body composition beyond BMI. Standard obesity metrics obscure critical heterogeneity: our findings advocate for routine imaging-based phenotyping in metabolic liver disease assessments.

Interestingly, certain parameters such as BMI and LDL were lower in the high-risk group (subtype 3) compared to the medium-risk group. This may reflect the phenomenon of metabolic burnout or sarcopenic obesity, where disease progression is accompanied by loss of lean mass or nutritional decline despite underlying metabolic dysfunction [21]. Additionally, the impact of intensive medical therapy—such as statin use or diabetes management—may contribute to lower lipid or weight values in this group [22]. These findings underscore the limitations of BMI and LDL as isolated markers in advanced stages of MAFLD and highlight the need for more nuanced assessments of metabolic risk.

Perhaps the most striking is the dominant predictive power of SVO for elevated ASCVD risk. Even after adjustment for classical risk factors (e.g., LDL, hypertension), SVO independently conferred a nearly fourfold increase in cardiovascular risk, exceeding that of diabetes or male sex. This positions SVO as a “cardio-hepato-metabolic” nexus, warranting integrated screening strategies that transcend organ-specific silos. Such an approach aligns with calls for multidisciplinary care models in MASLD and metabolic syndrome [6].

From a clinical standpoint, these findings advocate for incorporating SVO assessment into MASLD risk stratification algorithms, particularly in populations with elevated BMI or diabetes. The use of non-invasive, accessible tools such as bioelectrical impedance analysis (BIA) and MRI/MRE allows for scalable implementation. Identifying SVO therapeutic interventions, such as resistance training, protein-optimized nutrition, and insulin-sensitizing therapies—strategies shown to reverse both sarcopenia and hepatic steatosis [23].

In resource-limited settings, a pragmatic approach to screening for sarcopenic visceral obesity may involve combining BIA for muscle mass estimation with waist circumference as a proxy for visceral adiposity. This low-cost, scalable strategy offers a clinically viable alternative to imaging-based assessments, supporting integration of SVO screening into routine MASLD risk stratification and community-level metabolic surveillance.

A particularly novel element of this study is the development of a three-tier risk stratification model (Red, Yellow, Green), which integrates SVO status, fibrosis severity, and cardiovascular risk. This system highlights the multidimensional burden faced by patients with coexisting metabolic and hepatic dysfunction. The Red tier, characterized by 100% prevalence of F3–F4 fibrosis, elevated visceral fat, and reduced appendicular lean mass, identifies a distinct subgroup with overlapping fibrosing and atherogenic phenotypes. Notably, male sex and elevated ALT were independent predictors of Red-tier classification, reinforcing the gender-specific vulnerability observed in advanced MASLD, despite the overall higher prevalence of SVO in females. These findings resonate with recent calls for precision hepatology, emphasizing that phenotypic clustering—rather than isolated metrics—may more accurately reflect clinical risk and resource prioritization needs [24].

The tiered system also reflects a potentially scalable tool for front-line clinicians to triage patients by composite hepatic–cardiometabolic risk, especially in resource-constrained settings where access to elastography or liver biopsy is limited. The Yellow tier, defined by SVO with additional comorbidities, may represent a critical “window of opportunity” for intervention prior to irreversible fibrotic progression. Meanwhile, the Green tier still warrants close monitoring, as these patients—though lacking advanced fibrosis or immediate cardiovascular threats—harbor the underlying SVO.

This study offers several methodological and conceptual strengths that meaningfully advance the field of MASLD research. First, it is the first investigation in a Middle Eastern cohort to apply a rigorously defined SVO phenotype using the 2022 ESPEN/EASO sarcopenia consensus criteria combined with MRI-derived visceral fat quantification, providing precise and reproducible body composition profiling that avoids the limitations of BMI- or waist-based surrogates. Second, we incorporated magnetic resonance elastography (MRE) for fibrosis staging in a subset of patients, offering high-fidelity, non-invasive hepatic characterization and enhancing the internal validity of our fibrosis-related outcomes. Third, the study introduces a novel tier-based clinical risk stratification model that integrates SVO, fibrosis severity, and ASCVD risk into a pragmatic, phenotypically anchored framework. This tier model—comprising Red, Yellow, and Green categories—represents an original contribution that enables multidimensional risk assessment and may serve as a scalable tool for clinical triage and decision-making across hepatology, endocrinology, and primary care settings. In addition, this is among the first studies globally to propose that SVO—traditionally considered a metabolic complication—may act as a central organizing axis linking hepatic fibrogenesis and cardiovascular risk. By positioning muscle-fat imbalance at the heart of MASLD pathophysiology, this study shifts the paradigm toward integrated cardio-hepato-metabolic risk profiling. Finally, the inclusion of a diverse, real-world population from an underrepresented region enhances the generalizability of findings and supports equity in global MASLD research.

However, several limitations must be acknowledged. Based on a single-center Egyptian cohort, our findings may not fully extrapolate to global MASLD populations. Ethnic variation in genetic risk alleles (e.g., *PNPLA3* I148M), along with regional differences in diet, physical activity, and metabolic care, may influence disease expression. Validation in multi-ethnic, geographically diverse cohorts is essential to confirm generalizability and uncover population-specific determinants of MASLD. To validate SVO as a predictive marker, longitudinal studies are needed to assess its role in the progression of cirrhosis, cardiovascular events, and overall mortality. Standardized criteria and body composition imaging would further strengthen its clinical utility and integration into risk stratification models. Importantly, the absence of structured assessments of physical activity, dietary patterns, and cardiovascular parameters—such as resting heart rate and autonomic response to exertion—represents a key limitation in this study. These factors are integral to the regulation of body composition, hepatic lipid flux, and systemic cardiometabolic risk, and their omission constrains our ability to delineate the behavioral and physiological mechanisms underpinning the association between sarcopenic visceral obesity (SVO) and MASLD. Future investigations would benefit from the integration of validated lifestyle instruments and cardiometabolic markers to refine risk stratification and unravel the complex interplay between metabolic, muscular, and hepatic domains. Additionally, while MRI and BIA offer robust diagnostic capability, access remains limited in some low-resource settings.

In resource-limited settings, a pragmatic approach to screening for sarcopenic visceral obesity may involve combining BIA for muscle mass estimation with waist circumference as a proxy for visceral adiposity. This low-cost, scalable strategy offers a clinically viable alternative to imaging-based assessments, supporting integration of SVO screening into routine MASLD risk stratification and community-level metabolic surveillance.

*In conclusion*, SVO is a highly prevalent and clinically significant phenotype among MASLD patients, independently associated with advanced hepatic fibrosis and cardiovascular risk. Our findings call for routine screening for sarcopenia and visceral adiposity in MASLD management, particularly in women, patients with morbid obesity, and those with metabolic syndrome. Incorporating SVO into diagnostic and therapeutic pathways holds promise for improving outcomes in MASLD—a disease that demands multidimensional care.

## Data Availability

“Data is available upon request from the corresponding author.”

## Abbreviations

MASLD: Metabolic dysfunction-associated steatotic liver disease
SVO: Sarcopenic visceral obesity
SMA: Skeletal muscle area
ALM: Appendicular lean mass
BIA: Bioelectrical impedance analysis
MRI: Magnetic resonance imaging
MRE: Magnetic resonance elastography
VFA: Visceral fat area
BMI: Body mass index
WC: Waist circumference
FIB-4: Fibrosis-4 index
AST: Aspartate aminotransferase
ALT: Alanine aminotransferase
HOMA-IR: Homeostatic model assessment for insulin resistance
HbA1c: Hemoglobin A1c
LDL: Low-density lipoprotein cholesterol
TG: Triglycerides
ASCVD: Atherosclerotic cardiovascular disease
aOR: Adjusted odds ratio
OR: Odds ratio
CI: Confidence interval
HTN: Hypertension
DM: Diabetes mellitus
ESPEN: European Society for Clinical Nutrition and Metabolism
EASO: European Association for the Study of Obesity
WHO: World Health Organization
